# Genome-wide identification of calcium-dependent protein kinases in soybean and analyses of their transcriptional responses to insect herbivory and drought stress

**DOI:** 10.1038/srep18973

**Published:** 2016-01-06

**Authors:** Christian Hettenhausen, Guiling Sun, Yanbiao He, Huifu Zhuang, Ting Sun, Jinfeng Qi, Jianqiang Wu

**Affiliations:** 1 Department of Economic Plants and Biotechnology, Kunming Institute of Botany, Chinese Academy of Sciences, Kunming 650201, China

## Abstract

Calcium-dependent protein kinases (CDPKs) are plant-specific calcium sensors that play important roles in various aspects of plant physiology. Here, we investigated phylogenic relationships, chromosomal locations, gene structures, and tissue-specific, herbivory- and drought-induced expression profiles of soybean (*Glycine max*) *GmCDPK*s. Fifty *GmCDPK* genes were identified, which phylogenetically grouped into 4 distinct clusters and distributed across 13 sub-clusters. Individual classes of *GmCDPK*s harbor highly conserved mRNA splicing sites, and their exon numbers and lengths were consistent with the phylogenetic relationships, suggesting that at least 13 ancestral *CDPK* genes had emerged before the split of monocots and eudicots. Gene expression analysis indicated that several *GmCDPK*s were tissue-specific expressed. *GmCDPKs’* transcript levels changed after wounding, exhibited specific expression patterns after simulated *Spodoptera exigua* feeding or soybean aphid (*Aphis glycines*) herbivory, and were largely independent of the phytohormones jasmonic acid and salicylic acid. The most pronounced transcriptional responses were detected after drought and abscisic acid treatments with more than half of all *GmCDPKs* being upregulated, suggesting their important roles during abiotic stress responses in soybean. Our data provide an important foundation for further functional dissection of GmCDPKs, especially in the context of soybean-insect interactions and drought stress adaptation.

Ca^2+^ is a ubiquitous second messenger that is critical for signal transduction in eukaryotes by activating various cellular processes in response to developmental and stress stimuli^1^,^2^,^3^. In plants, transient Ca^2+^ changes in the cytoplasm are sensed and decoded by an array of calcium sensors, such as calmodulins, calmodulin-binding proteins, calcineurin B-like proteins, and calcium-dependent protein kinases (CDPKs or CPKs), and these Ca^2+^ sensors further interact with their downstream target proteins and in turn trigger Ca^2+^ signature-specific responses.

CDPKs have been identified throughout the plant kingdom and in some protozoans, but not in animals or fungi. A typical CDPK consists of five domains: a variable N-terminal domain, a protein kinase domain, an autoinhibitory junction domain, a calmodulin-like domain, and a C-terminal domain. The N-terminal domain shows the highest sequence divergence among CDPKs and often contains myristoylation or palmitoylation sites that are believed to be associated with subcellular targeting^4^. The protein kinase domain is the catalytic domain with an ATP binding site, and is adjacent to the autoinhibitory junction domain^5^ and the consecutive calmodulin-like domain that contains EF hands for calcium binding^4^. The C-terminal domain is also variable and differs in lengths and amino acid compositions amongst CDPKs. It has been suggested that the N- and C-terminal variable domains determine the specific function of individual CDPK^6^. Binding of Ca^2+^ to the calmodulin-like domain results in conformational changes that in turn displace the autoinhibitory domain from the kinase domain, and thereby activate CDPKs.

Recent findings have pointed to the roles of CDPKs in wounding- and herbivory-induced plant responses. The expression and enzymatic activity of maize (*Zea mays*) ZmCDPK11 are up-regulated by wounding, linolenic acid, and methyl jasmonate (MeJA), and the wound-induced activation of ZmCPK11 is dependent on phosphatidic acid produced in the phospholipase D (PLD) pathway^7^,^8^. Arabidopsis AtCPK3 and AtCPK13 phosphorylate the transcription factor (TF) HsfB2a, and HsfB2a in turn regulates the transcript accumulation of several stress-related genes after *Spodoptera littoralis* feeding^9^. Tomato (*Solanum lycopersicum*) LeCPK1 inhibits the plasma membrane H^+^-ATPase to induce extracellular alkalization as a part of the wound and pathogen defense signaling cascades^10^, and LeCDPK2 phosphorylates LeACS2 in response to wounding, stabilizing this rate-limiting enzyme for ethylene (ET) biosynthesis by direct phosphorylation^11^. In addition, Zhang *et al.* showed that in grape wine, several *CDPK* genes’ expression was increased by ET-treatment^12^. *N. attenuata* CDPKs, NaCDPK4 and NaCDPK5, play redundant and negative roles in mediating wounding- and herbivory-elicited JA accumulations, and silencing *NaCDPK4* and *NaCDPK5* greatly elevates plant resistance to the specialist insect *Manduca sexta*^13^,^14^.

Drought stress poses another major threat to plants and recent research has shown that CDPKs play important roles in regulating plant adaptation to drought. The transcript levels of *ZmCPK12* are highly induced by drought in maize seedlings, and overexpression of *ZmCPK12* in Arabidopsis improves plant survival under drought conditions^15^. Similarly, *Populus euphratica PeCPK10* confers drought tolerance and enhances the expression of several ABA-responsive genes when being overexpressed in Arabidopsis^16^. OsCPK9 and OsCDPK7 positively regulate drought stress tolerance in rice, most likely by affecting the transcript levels of ABA- and stress-responsive genes^17^,^18^. Importantly, guard cell-expressed Arabidopsis CPK21 was identified as a major interacting partner of the guard cell anion channel SLAC1 and thus regulates stomatal ABA signal transduction^19^.

CDPKs constitute a large multigene family in higher plants with 34, 35, 31, 30, and 26 CDPK genes found in Arabidopsis, maize, rice (*Oryza sativa*), poplar (*Populus trichocarpa*), and wheat (*Triticum aestivum*), respectively^4^,^20^,^21^,^22^,^23^. Soybean (*Glycine max*) is one of the most important crop plants worldwide, providing oil and protein to human and livestock. Valmonte *et al.* identified 47 CDPK genes from soybean and revealed extensive sequence conservation amongst them^24^. Soybean CDPKα and CDPKγ phosphorylate a serine acetyltranferase involved in cysteine biosynthesis after oxidative stress^25^. In soybean nodules, certain CDPKs phosphorylate the membrane protein nodulin-26 affecting its voltage-sensitive channel activity^26^, and nodulin-100, a sucrose synthase that is essential for the cleavage of sucrose translocated from the shoots to the roots in support of nodule C/N-metabolism^27^,^28^. However, still little is known about the roles of GmCDPKs in soybean development, growth, and in adaptation to biotic and abiotic stresses.

In this study, we carried out a genome-wide analysis and identified 50 *GmCDPK*s. Phylogenetic analysis was performed and *GmCDPK*s gene structures were compared to reveal their evolutionary relationships. Furthermore, we examined the expression of all *GmCDPK*s after wounding, simulated *Spodoptera exigua* herbivory, *Aphis glycines* feeding, and treatments of JA, ET, and SA, phytohormones important for plant defense against insects, as well as in response to drought and ABA. We found that the transcript levels of many *GmCDPK*s were not dependent on JA or SA signaling but were specifically altered in response to herbivory, suggesting that GmCDPKs may play important roles in soybean defense against insects. Moreover, almost 80% of all *GmCDPKs* were induced after drought or ABA treatments, highlighting their important role in regulating drought stress responses.

## Results

### Identification and characteristics of *GmCDPK* genes

To find all CDPKs in soybean, a domain search against all predicted proteins in the soybean genome (http://www.phytozome.net/soybean) was performed. A total of 68 proteins containing a protein kinase domain and at least one EF hand domain were identified, amongst which 5 showed high similarities to CRKs, CaMKs, and CCaMKs and were thus eliminated, and the remaining proteins were designated as GmCDPKs. Further database search using these GmCDPKs as queries against the draft genome and predicted mRNAs resulted in no further hits. In total, 63 proteins, including the alternatively spliced forms, from 50 gene loci were identified as GmCDPKs, and their coding genes were designated as *GmCDPK1* to *GmCDPK50*, according to their locations on soybean chromosomes (Supplementary Table S1). The alternatively spliced regions in *GmCDPK1*, *GmCDPK9*, *GmCDPK35* and *GmCDPK46* contain the EF hand domains, resulting in protein isoforms with less than 4 EF hands (Supplementary Table S1). *GmCDPK50* lacked ~600 bp at the 5′ end, which normally accounts for the variable N-terminal domain and a part of the protein kinase domain. We inspected the 5′ intergenic region (about 4 kb) and did not find any fragment encoding the conserved protein kinase domain. These findings strongly indicate that *GmCDPK50* is a pseudogene.

Myristoylation and palmitoylation at the N-terminal regions of CDPKs are correlated with their subcellular localization and substrate specificity^29^,^30^,^31^. Forty-nine of the 63 GmCDPK proteins possess myristoylation sites that are, with the exception for GmCDPK2, GmCDPK38, and GmCDPK41, located in the first 32 amino acids. Seven of the 63 GmCDPK proteins have no the palmitoylation sites. Most palmitoylation sites are located within the first 7 amino acids, albeit those of 7 GmCDPKs were predicted to be located close to the 50th amino acid of the N-terminus (Supplementary Table S1). We further predicted the organelle-specific localization using TargetP and NucPred^32^, and found 10 GmCDPKs to be localized in chloroplasts, and that 2 GmCDPKs, GmCDPK29 and GmCDPK49, contain mitochondrion-specific targeting sequences (Supplementary Table S1). GmCDPK37.2 and GmCDPK41 were predicted to be located in the nucleus.

### Phylogenetic relationships, gene structures, and potential functional divergence of GmCDPKs

**Figure 1:**
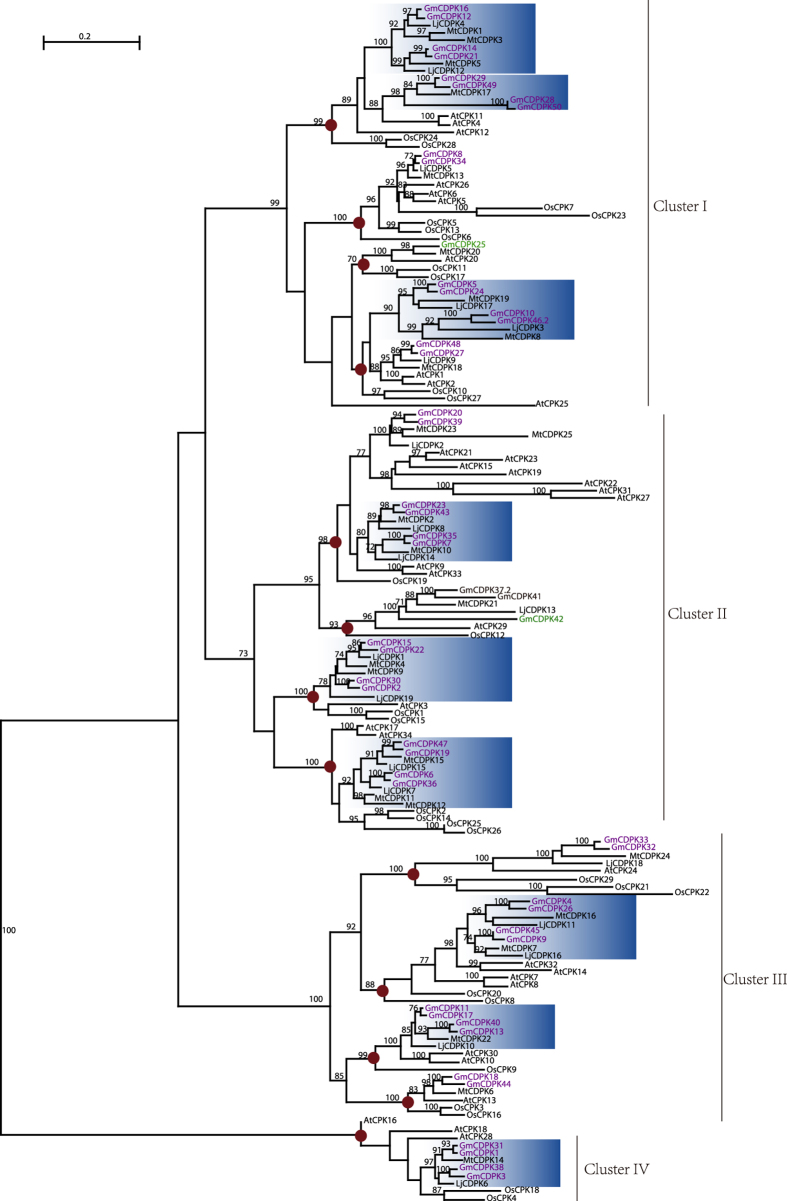
Phylogenetic relationship of soybean, Arabidopsis, *Lotus japonicas*, *Medicago truncatula*, and rice CDPKs. A phylogenetic tree was constructed for 50 soybean (*Glycine max*), 19 *Lotus japonicas*, 25 *Medicago truncatula,* 29 rice (*Oryza sativa*), and 34 *Arabidopsis thaliana* CDPKs. The 4 phylogenetic clusters designated as I–IV are marked with vertical bars. Thirteen sub-clusters containing both eudicots and monocots are highlighted with brown dots. The legume *CDPKs* that probably resulted from the genome duplication event (GDE) occurred in the legume common ancestor are shaded in blue. The genes retained after the soybean GDE are in purple letters, except that GmCDPK25 and GmCDPK42 without paralogs are in green and the paralog pair, GmCDPK37 and GmCDPK41 (88% sequence identity) that is not from the soybean GDE, is in black. Numbers above branches show bootstrap support values from maximum likelihood analyses.

To gain insights into the evolution of GmCDPKs, we also identified the *CDPK* genes from two other legume species, *Lotus japonicas* and *Medicago truncatula*, using the same procedure and found 19 and 25 *CDPK* genes, respectively (Supplementary Table S2). A maximum likelihood tree including all CDPKs from these three legumes, Arabidopsis, and rice was constructed (Fig. 1). The phylogenetic analysis indicated that, consistent with the previous reports^20^,^24^,^33^, all CDPKs from these 5 species grouped into 4 major clusters (Fig. 1, Clusters I–IV). Detailed inspection revealed that the CDPKs are distributed among 13 sub-clusters (Fig. 1, indicated by the brown dots), each containing CDPKs from eudicots and monocots. Therefore, at least 13 ancestral *CDPK* genes existed prior to the split of eudicots and monocots. As for the 3 legume species, 9 out of the 13 sub-clusters (Fig. 1, blue background) contain at least 2 paralogous CDPKs of *L. japonicas* and *M. truncatula* and 4 paralogs of soybean. The paralogs in these 9 sub-clusters probably resulted from the genome duplication event that occurred in the common ancestor of legumes and the consequent genome duplication that occurred uniquely in soybean^34^. Notably, GmCDPK25 clustered as a singleton in Cluster I and GmCDPK37/41/42 grouped as a triad in Cluster II (Fig. 1 and Supplementary Fig. S1), suggesting that gene loss may have had occurred after the genome duplication events.

To correlate the phylogenetic relationships with the patterns of *GmCDPK* gene structures, we analyzed the intron-exon characteristics of *GmCDPK*s. The exon numbers and lengths within all 13 sub-clusters are consistent with their phylogenetic relationships, although the intron lengths are variable (Supplementary Fig. S1). The median length of all introns is 265 bp, the minimum is 31 bp in *GmCDPK41*, and the maximum is 4168 bp in *GmCDPK6*. Most *GmCDPK*s in Clusters I-III have 6 to 9 exons, while *GmCDPK*s in Cluster IV have 12 or 14 exons. The complex gene structures of Cluster IV *GmCDPK*s suggest a different evolutionary history and probably functional divergence from the other *GmCDPK*s.

### Chromosomal distributions and development-related expression profiles of *GmCDPK*s

**Figure 2:**
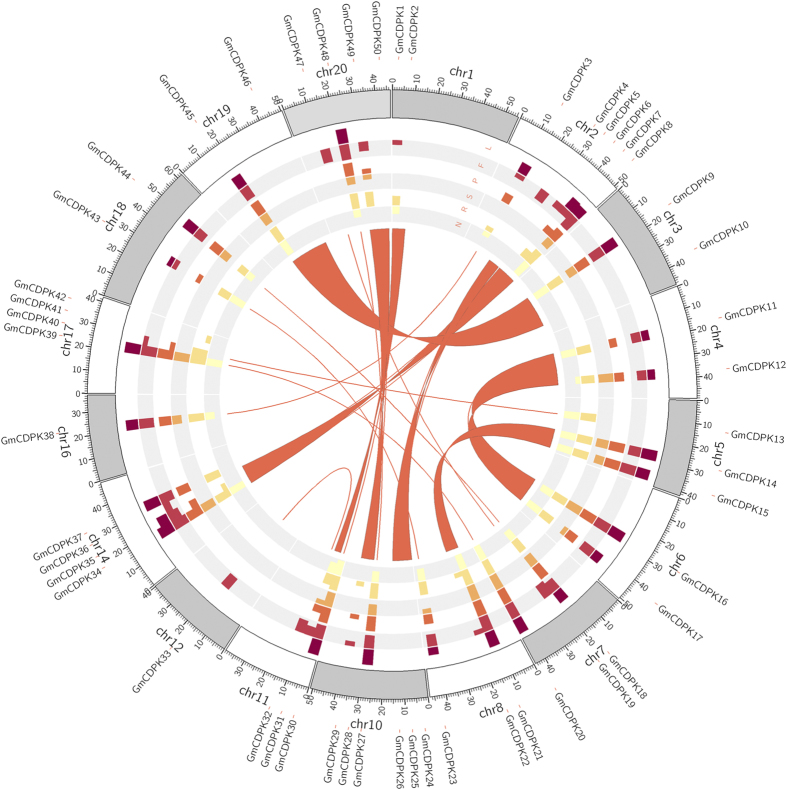
*GmCDPK*s’ Chromosome distributions, development-related expression profiles, and paralogous relationships resulted from the soybean genome duplication event. The distribution of *GmCDPK* genes on 17 soybean chromosomes are given in the outer circle (the 3 chromosomes containing no *GmCDPK*s are not shown), where the numbers represent the chromosome length in Mb. *GmCDPK*s’ expression levels in 6 different tissues and developmental stages are displayed as filled blocks in yellow to dark red, with the block height proportional to the expression level (LOG_2_ transformed) (L, young leaves (0.4 Leaflets unfurled); F, flowers; P, one cm pods; S, seeds 14 days after flowering; R, roots; N, nodules). GmCDPK pairs with sequence identities more than 94%, which had most likely derived from the recent soybean genome duplication (SGD), are connected by orange arcs in the inner circle. Their adjacent chromosomal regions that had derived from SGD are indicated by arcs filled with orange. For the convenience of display, the localization of *GmCDPK* genes on the chromosomes is disproportional to their real positions.

At least two genome duplication events occurred in the evolutionary history of soybean, which were followed by numerous chromosome rearrangements, gene structure diversification, and multitudinous gene loss, and these events led to rather complex gene families. Among the 20 chromosomes, chromosomes 9, 13, and 15 carry no *GmCDPK*s, chromosomes 12 and 16 each contain only 1 *GmCDPK* gene, chromosomes 2 and 10 each contain 6 *GmCDPK* genes, and all other chromosomes have 2–4 *GmCDPK*s (Fig. 2). All *GmCDPK*s, except *GmCDPK26*, are located within the chromosome ends, in agreement with the data from soybean genome sequencing: ~78% of all soybean genes were predicted to be located in these regions^34^. At least 7 segment duplications contributed to the expansion of the *GmCDPK* gene family (Fig. 2 and Supplementary Fig. S1). For example, *GmCDPK1* is adjacent to *GmCDPK2* on Chromosome 1 and *GmCDPK31* is close to *GmCDPK30* on Chromosome 11; *GmCDPK1* and *GmCDPK31* are paralogs, as are *GmCDPK2* and *GmCDPK30* (Fig. 2). Inspection of the genes between the individual *CDPK*s showed high similarity between the two chromosomal regions, suggesting that they originated from segment duplication. Another case connects Chromosome 10 and Chromosome 20: It has been reported that the long arms of Chromosome 10 and Chromosome 20 very likely derived from segment duplication^34^; supporting this scenario, *GmCDPK28* on Chromosome 10 and *GmCDPK49* on Chromosome 20 showed high sequence identity, and so did their respective adjacent genes *GmCDPK29* and *GmCDPK50* (Fig. 2 and Supplementary Fig. S2). The physical distance between *GmCDPK41* and *GmCDPK42* on Chromosome 17 is ~ 3 kb, and their sequence identity is 68% which suggests that these two genes may have originated from a tandem duplication event.

A gene’s function is often correlated with its transcriptional profile. To gain insights into the potential functions of *CDPK* genes in soybean development and growth, RNA-Seq data from expression profiles of 6 different soybean tissues and developmental stages obtained from SoyBase^35^ were analyzed and aligned with the phylogeny of the 50 GmCDPKs (Fig. 2). To determine potential functional divergence, we divided the paralogous genes into two types - those derived from the early and those from the later genome duplication event respectively, according to their clustering in the phylogenetic tree and their sequence identities. Most paralogous genes from the later genome duplication event, which clustered as sister branches in the phylogenetic tree (Fig. 1) and shared sequence identities no less than 94% (Supplementary Fig. S1), presented largely similar expression patterns. Notably, *GmCDPK47* and *GmCDPK19*, *GmCDPK6* and *GmCDPK36*, and *GmCDPK32* and *GmCDPK33* were almost exclusively expressed in flowers, and *GmCDPK13* and *GmCDPK40* were highly expressed in roots, suggesting that they may be involved in these organs’ development and growth (Fig. 2). However, some paralogous *GmCDPK*s exhibited rather different patterns of expression. *GmCDPK22* and *GmCDPK2* had very low expression, while their paralogs (*GmCDPK15* and *GmCDPK30*, respectively) appeared to have relatively high expression throughout the whole sample set; *GmCDPK28* and *GmCDPK50* showed very low expression across all 6 samplings, whereas their paralogous counterparts from the same tetrad (77% sequence identity), *GmCDPK29* and *GmCDPK49*, were transcribed in flowers, seeds, and roots (Fig. 2). In contrast, the majority of paralogous genes from the early genome duplication event clustered as tetrads with sequence identities lower than 94% and showed distinct transcriptional profiles (Supplementary Fig. S2).

### *GmCDPK*s transcriptional response to wounding and herbivore feeding

**Figure 3:**
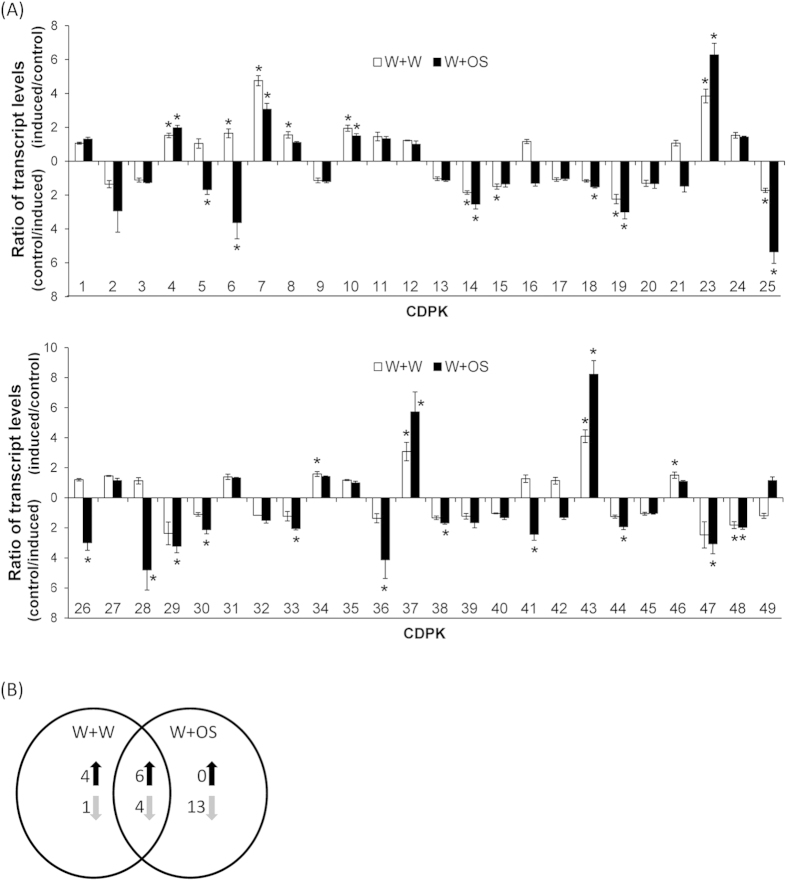
Wounding and simulated herbivory change the transcript levels of various *GmCDPK*s. Plants were wounded with a pattern wheel, and 20 μL of water (W+W) or *S. exigua* oral secretions (W+OS) were immediately applied to the wounds; untreated plants served as controls. Individual leaves from 5 replicate plants were harvested 1.5 h after the treatments, and *GmCDPK*s’ expression levels were analyzed by q-PCR. (**A**) Ratios (mean ± SE) between the *GmCDPK* transcript levels (Log_2_ transformed) in W+W- or W+OS-treated and untreated controls. Asterisks indicate significant changes (*t*-test, p < 0.05) of more than 1.5-fold up- or down-regulated genes. (**B**) Venn diagram depicting the number of genes having similar or differential (*t*-test, p < 0.05) expression in response to W+W and W+OS treatments.

Several CDPKs have been demonstrated to be involved in plant-herbivore interactions^9^,^13^. *S. exigua* is a generalist insect herbivore that causes damages to soybean. To gain insights into the roles of *GmCDPK*s in soybean response to *S. exigua* herbivory, leaves were wounded with a pattern wheel, and the generated puncture wounds were immediately treated with water (W+W) or *S. exigua* oral secretions (W+OS) to cause mechanical damages or to simulate herbivore feeding (the feeding behavior of *S. exigua* cannot be synchronized, thus simulated herbivory was used). Given that the transcript levels of *CDPK*s usually reach their highest 1.5 h after these treatments^13^,^36^ (and see below), samples were harvested 1.5 h after treatments and the expression levels of *GmCDPK*s were analyzed by q-PCR (quantitative real-time PCR). Mechanical wounding significantly increased the transcript abundances of 10 out of 48 *GmCDPK*s (the truncated and the putatively pseudogenized *GmCDPK22* and *GmCDPK50* were excluded from further analyses), whereas 5 *GmCDPK*s showed decreased transcript levels (Fig. 3A). In contrast, W+OS treatment only increased the expression of 6 *GmCDPK*s, whereas 17 *GmCDPK*s’ transcript levels were suppressed (Fig. 3A). Notably, *GmCDPK*s whose levels were upregulated by W+OS treatment also showed elevated levels after W+W, and 4 out of 5 *GmCDPK*s with decreased expression levels after wounding were also suppressed following W+OS treatment (Fig. 3B). Compared with their levels after W+W treatment, greater (about 2-fold) transcription levels of *GmCDPK23* and *GmCDPK43* were detected after W+OS treatment; in contrast, 10 *GmCDPK*s (*GmCDPK6*, *7*, *25*, *26*, *28*, *30*, *36*, *41*, *44* and *47*) had significantly (*t*-test, p < 0.05) lower expression levels (about 6-fold for *GmCDPK6* and *GmCDPK28*) after induction with W+OS compared to W+W treatments. Notably, *GmCDPK23* and *GmCDPK43* are paralogs and share high sequence identity (97% on the protein level). Similarly, *GmCDPK6* and *GmCDPK36* are paralogous genes (98% identical) and both genes were suppressed by W+OS treatment. In contrast, *GmCDPK4* and *GmCDPK26*, as well as *GmCDPK37* and *GmCDPK41*, may have also derived from gene duplications (95 and 88% sequence identity, respectively) but these pairs responded in opposite ways to W+OS treatment (Fig. 3A).

**Figure 4:**
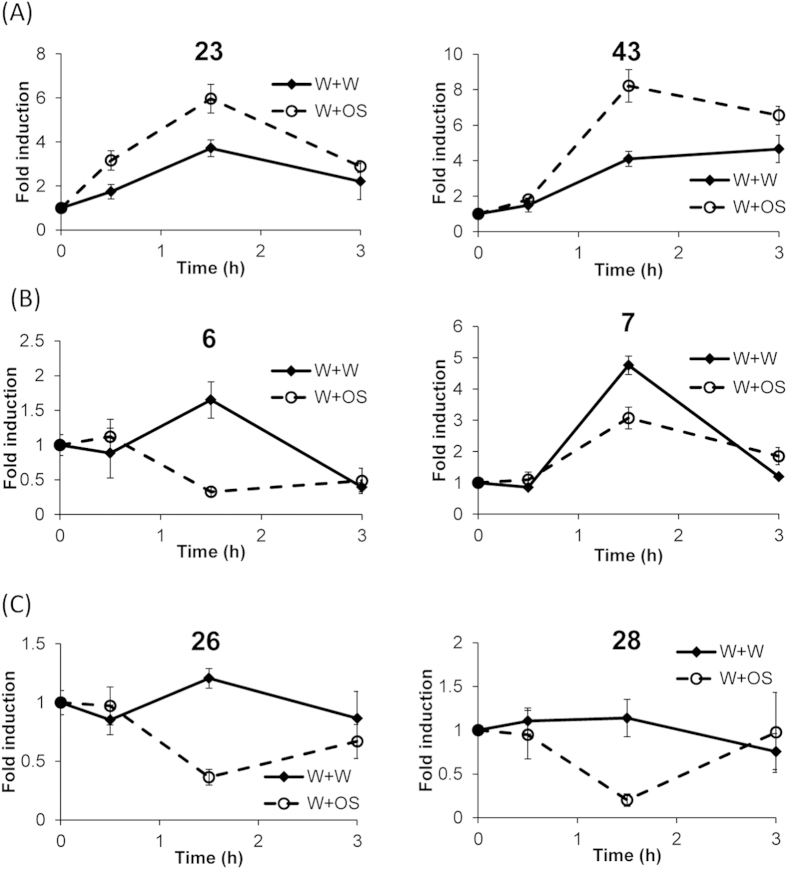
Transient changes of *GmCDPK* transcript levels following wounding and simulated herbivory. Plants were wounded with a pattern wheel, and 20 μL of water (W+W) or *S. exigua* oral secretions (W+OS) were immediately applied to the wounds; untreated plants served as controls. Individual leaves from 5 replicate plants were harvested at the indicated times. Mean (±SE) transcript levels (LOG2 transformed) of *GmCDPK23* and *GmCDPK43* (**A**), *GmCDPK6* and *GmCDPK7* (**B**), and *GmCDPK26* and *GmCDPK28* (**C**) were normalized to their levels in untreated controls.

W+W and W+OS treatment induced distinct expression patterns of *GmCDPK6*, *7*, *23*, *43*, *26* and *GmCDPK28* (Fig. 3A). To further investigate the transcriptional responses of *GmCDPK*s to simulated *S. exigua* herbivory, these genes were selected and analyzed in detail in samples harvested 0.5, 1.5, and 3 h after elicitations. All these *GmCDPK*s showed the highest differences compared to untreated controls 1.5 h after the elicitations and the transcript levels mostly returned to the uninduced state 3 h after the initial treatments. *GmCDPK23* and *GmCDPK43* were up-regulated after both W+W and W+OS treatment, and were higher expressed after W+OS than after W+W treatment (Fig. 4A). W+W treatment induced the expression levels of *GmCDPK6* and *GmCDPK7*, but adding OS to wounds suppressed the wounding-induced responses (Fig. 4B). While W+W barely changed the levels of *GmCDPK26* and *GmCDPK28*, W+OS repressed their transcript levels (Fig. 4C).

**Figure 5:**
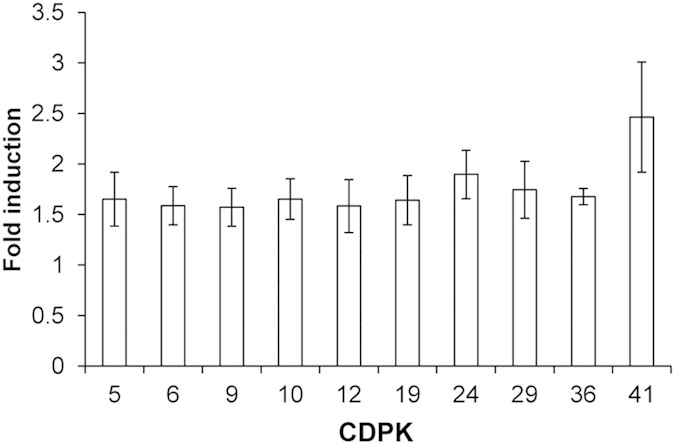
Aphid (*Aphis glycines*) herbivory-induced changes in *GmCDPK*s’ transcript abundances. 10 nymphs and 10 adult aphids were placed together on leaves of the second trifoliate and leaves were immediately enclosed between two 50 mL food-quality plastic containers secured with miniature claw-style hair clips. Uninfested enclosed leaves served as controls. *GmCDPK* expression was measured with q-PCR in 6 replicated samples collected 8 h after the start of the treatment. Values represent significantly (*t*-test, p < 0.05) and more than 1.5-fold induced *GmCDPK* transcripts normalized to their respective expressions in control samples (mean ± SE); *GmCDPK*s whose expression levels did not change are not shown.

Next, we investigated whether not only chewing herbivores but also aphids, as piercing-sucking feeders, could affect *GmCDPK*s. Leaves were exposed to 10 nymphs and 10 adult soybean aphids, *Aphis glycines*, and after feeding for 8 h, samples were collected and *GmCDPK* transcript levels were analyzed by q-PCR. Ten *GmCDPK*s (*GmCDPK9*, *10*, *12*, *19*, *29*, *41*, and the paralogs *GmCDPK5* and *GmCDPK24*, as well as *GmCDPK6* and *GmCDPK36*) exhibited significantly increased transcript levels (Fig. 5). In contrast to W+W and W+OS treatments, no *GmCDPK*s were negatively regulated. Notably, the expression levels of *GmCDPK6* and *GmCDPK36* were suppressed by W+OS treatment but increased after *A. glycines* herbivory, and this pattern was also found for *GmCDPK5*, *GmCDPK29* and *GmCDPK41*, suggesting that these *GmCDPK*s may function very differently in soybean defense against *S. exigua* and *A. glycines*.

### JA-, SA-, and ethylene-induced transcriptional changes

**Figure 6:**
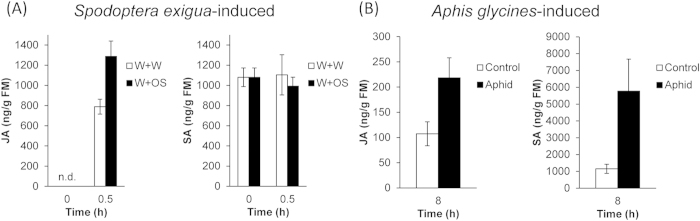
Herbivory-induced changes in JA and SA levels. (**A**) Hormones induced by wounding and simulated *S. exigua* herbivory. Plants were wounded with a pattern wheel, 20 μL of water (W+W) or *S. exigua* oral secretions (W+OS) were immediately applied to the wounds, and the JA and SA contents were measured in samples from 5 replicated plants collected 0.5 h after treatments. Non-treated plants served as controls. (**B**) *A. glycines*-induced hormone levels. 10 nymphs and 10 adult aphids were placed together on leaves of the second trifoliate and leaves were immediately enclosed between two 50 mL food-quality plastic containers secured with miniature claw-style hair clips. Uninfested enclosed leaves served as controls. Samples were harvested after 8 h, and their JA and SA contents (mean ± SE; N = 6) were analyzed on a high-performance liquid chromatograph-tandem mass spectrometer (HPLC-MS/MS).

Plant hormones JA, SA, and ET play central roles in plant-herbivore interactions by regulating a large number of genes related to plant defense responses. We determined hormonal changes after W+W and W+OS treatments and after aphid herbivory. By 30 min, W+W- and W+OS-treated leaves accumulated 790 and 1,288 ng/g of JA, respectively, indicating that soybean recognized certain elicitors in *S. exigua* OS and accumulated higher levels of JA to counteract *S. exigua* attack (Fig. 6A). In contrast to W+W and W+OS treatments, 8 h aphid feeding highly increased SA levels from 1,150 ng/g to 5,770 ng/g, but the JA contents were only slightly elevated (Fig. 6B).

**Figure 7:**
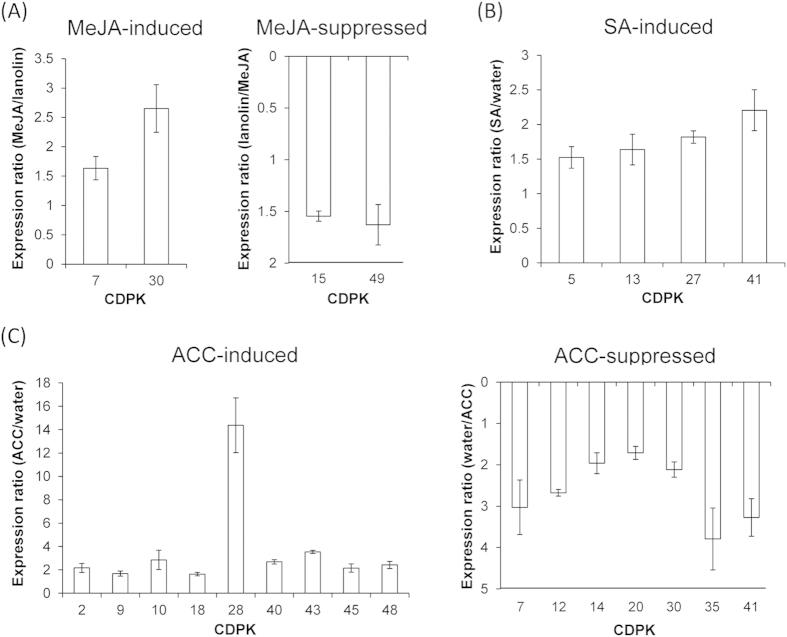
MeJA-, SA- and ET-induced changes in *GmCDPK* transcription. *CDPK* expression was measured in leaves from five replicate plants at 8 h post-treatment. Leaves were treated with 150 μg MeJA in lanolin paste, with lanolin as control (**A**) or were sprayed with either 1 mM SA (**B**) or 1 mM ACC (**C**) with water as control. Values represent mean ( ± SE; N = 5) expression ratios of significantly (*t*-test, p < 0.05) and more than 1.5-fold up- or down-regulated *GmCDPK* transcript levels normalized to the *GmCDPK* levels in the respective controls; *GmCDPK*s whose expression levels did not change are not shown.

To study whether herbivory-induced JA, SA, and ET play a role in modulating the transcript levels of *GmCDPKs*, we analyzed *GmCDPK* transcript accumulations in plants that had been treated with methyl jasmonate (MeJA) that readily diffuses into plants to release JA and SA or 1-aminocyclopropane-1-carboxylic acid (ACC; the precursor of ET), which were sprayed onto the leaves. Only a few *GmCDPK*s showed significantly altered transcript levels after JA and SA treatments: In contrast to W+W and W+OS treatment, supplementation of MeJA only elevated the levels of *GmCDPK7* and *GmCDPK30*, while two other genes, *GmCDPK15* and *GmCDPK49*, showed reduced expression levels (Fig. 7A). *GmCDPK5*, *13*, *27*, and *GmCDPK41* were slightly elicited by SA, but SA did not down-regulate any *GmCDPK*s (Fig. 7B). ET treatment induced more extensive transcriptional changes, with 9 up- and 7 down-regulated *GmCDPK*s. Amongst them, the paralogous *GmCDPK9* and *GmCDPK45* were upregulated and the pair *GmCDPK7*/*GmCDPK35* was down-regulated after ET treatment. Interestingly, *GmCDPK 7* and *30*, which were down-regulated by W+OS but induced by MeJA, were suppressed by ACC treatment, indicating that OS-induced ET may dominate the transcriptional responses for these two *GmCDPKs*. In contrast, *GmCDPK28* that showed the strongest induction by ET (14-fold; Fig. 7C) was 5-fold down-regulated after W+OS treatments and neither responded to JA nor SA, implying additional regulators of *GmCDPK28* expression following *S. exigua* herbivory. Overall, 38 out of 48 *GmCDPK*s showed transcriptional responses to herbivory or herbivore-related hormone treatments, underlining the important roles of GmCDPKs in plants responses to insect feeding (Supplementary Fig. S3).

### *GmCDPK* expression profiling after drought and ABA treatment

**Figure 8:**
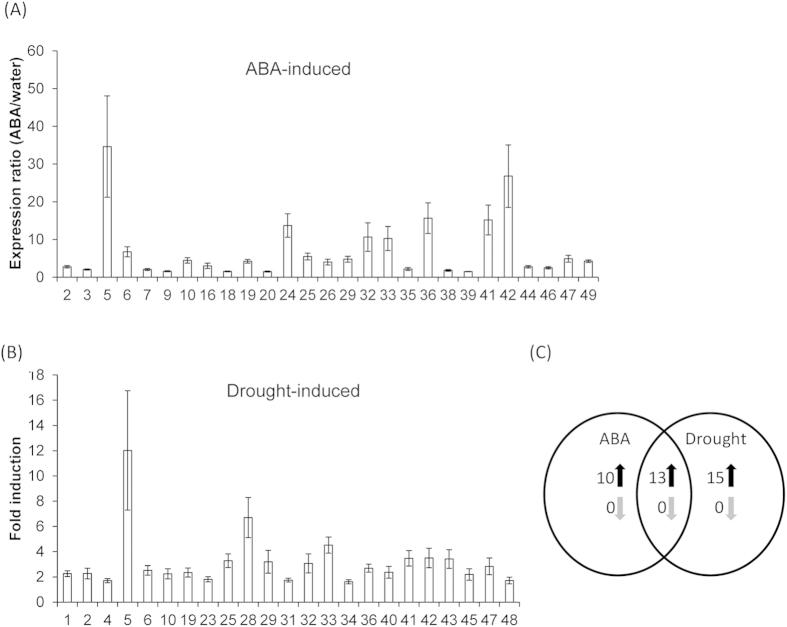
Drought- and ABA-induced changes in *GmCDPK* transcript levels. (**A**) Leaves were sprayed with 1 mM ABA or water as control and the transcript levels of *GmCDPK*s were measured in leaves from five replicate plants at 8 h post-treatment. (**B**) For drought treatments, watering was stopped for 5 days and samples were harvested when leaves begun to lose turgor and wilted. Values represent mean ( ± SE; N = 5) expression ratios of significantly (*t*-test, p < 0.05) and more than 1.5-fold up- or down-regulated *GmCDPK* transcript levels normalized to the *GmCDPK* levels in the respective controls; *GmCDPK*s whose expression levels did not change are not shown. (**C**) Venn diagram illustration of overlapping and specific transcriptional responses after ABA and drought treatments. Numbers show significantly (*t*-test, p < 0.05) and more than 1.5-fold up- or down-regulated genes in the respective group.

To gain insight into the functions of *GmCDPK*s in abiotic stress responses, we investigated the patterns of *GmCDPK*s’ expression after ABA and drought treatments (Fig. 8). With 23 and 27 up-regulated *GmCDPKs* respectively, about half of the *GmCDPKs* responded to the individual treatments and only 10 were not regulated by at least one of them. As expected, the transcriptional responses partially overlapped (Fig. 8C) and many paralogous *GmCDPKs*, such as *GmCDPK19* and *GmCDPK47* and *GmCDPK6* and *GmCDPK36* showed consistent expression patterns. These findings confirm the importance of CDPKs in drought stress and suggest that the transcriptional regulation of *GmCDPKs* in response to drought is of ancient nature and existed long before the recent soybean genome duplication event.

### Promoter *cis*-elements analysis of *GmCDPK*s

Promoter *cis*-elements play critical roles in the initiation of gene expression. Thus, bioinformatic analysis was done to identify possible *cis*-elements in the promoter sequences of *GmCDPK*s (Supplementary Dataset 1). Consistent with the proposed roles of CDPKs to be involved in multiple signaling pathways, most *GmCDPK*s carry several *cis*-elements related to stress responses; furthermore, most paralogs only showed moderate consistency in distributions of the *cis*-elements, indicating that after genome duplication events the promoters of the paralogous GmCDPKs have diverged (Supplementary Dataset 1). Notably, among the 48 *GmCDPK* promoters, 38 possess the HSE element (heat stress responsiveness), and 38 contain the TC-rich repeats (defense and stress responsiveness). Most of the ABA- or drought-inducible *GmCDPK* genes were found to contain ABRE (abscisic acid responsiveness) and/or MBS (MYB binding site involved in drought-inducibility) elements in their promoters. However, even SA treatment induced only 4 *GmCDPK*s, 37 *GmCDPK* promoters carry the TCA-elements (SA-responsive), and even 15 genes responded to wounding, only the *GmCDPK43* promoter consists of a WUN-motif (wound-responsive). *cis*-Element analysis also did not well predict the responsiveness of *GmCDPK*s to ET. More research is needed for a better understanding of transcriptional regulation in soybean plants, including TFs and their specific *cis*-elements.

## Discussion

A genome-wide database search, based on conserved domains and sequence similarities to known CDPKs, revealed 50 *GmCDPK* genes in the soybean genome. Thus we identified 3 additional *GmCDPK* genes, *GmCDPK2*, *GmCDPK24*, and *GmCDPK50*, compared with the *GmCDPK*s report by Valmonte *et al.*^24^. Similar to those in wheat^21^, all *GmCDPK* genes encoded at least one form of CDPK protein with 4 EF hands; in contrast, Arabidopsis^4^, rice^20^, and maize^22^ have CDPKs with less than 4 EF hands.

The pattern of *GmCDPK* chromosomal distribution is caused by two recent whole-genome duplication events, the early-legume duplication and the soybean-lineage-specific duplication, and subsequent chromosomal rearrangements and segment losses^37^. Most closely related homologs are not physically adjacent to each other or are even located at different chromosomes, indicating that segment duplication or genome rearrangement occurred during soybean evolution. We found evidence for two tandem duplication events: 1) *GmCDPK28* and *GmCDPK29* on Chromosome 10 are tandem duplicates and so are their respective paralogs *GmCDPK49* and *GmCDPK50* on Chromosome 20; 2) another tandem duplication event and consecutive segment duplication originated the homologs *GmCDPK41* and *GmCDPK42* on Chromosome 17 and *GmCDPK37* on Chromosome 14 but the paralog of *GmCDPK42* was lost over time.

Wounding regulates *CDPK* expression in several plant species^38^,^39^. However, changes in *CDPK* expression levels after herbivore challenges were only reported in the wild tobacco *N. attenuata,* in which 4 *NaCDPK*s were induced by wounding or adding *M. sexta* OS to wounds^13^,^36^. Plants recognize insect-specific factors to induce specific defense responses. *S. exigua* OS contain various fatty acid-amino acid conjugates (FACs)^40^, which are the best studied group of elicitors in lepidopteran insect OS^41^. Adding different synthetic FACs to wounded soybean leaves highly elicits ET and JA production^42^. Thus, it is likely that the FACs in the *S. exigua* OS are among the elicitors that induced OS-specific transcriptional changes of *GmCDPK*s, although other elicitors, such as proteins, cannot be ruled out. Feeding from a single-cell type, the phloem sieve element, aphids induce defenses in host plants which are distinct from those induced by chewing insects^43^. Consistently, the *GmCDPK* expression profiles induced by simulated *S. exigua* feeding and aphid treatment overlapped very little, and some *GmCDPK*s even exhibited opposite patterns: *GmCDPK5*, *6*, *36*, and *GmCDPK41* were suppressed by simulated *S. exigua* herbivory but induced after *A. glycines* feeding (Figs. 3 and 5).

Some CDPKs are known to function up- or downstream of hormone signaling to regulate plant wound- and herbivore-elicited defense responses. *S. exigua* feeding induced high levels of JA in soybean leaves (Fig. 6A); however, our analysis indicated that the transcriptional changes of almost all *GmCDPK*s were independent of JA signaling. *GmCDPK7* was wound- and also MeJA-induced but this response was likely not from JA alone, because after W+OS treatment, which elicited higher JA levels than did W+W, *GmCDPK7* transcript levels were down-regulated. Similarly, *GmCDPK30* was suppressed after W+OS treatment but induced by MeJA. Moreover, compared with simulated *S. exigua* herbivory, MeJA treatment only weakly changed *GmCDPK*s’ expression levels, suggesting that transcriptional regulation of most *GmCDPK*s is downstream of perception of wounding or *S. exigua* herbivory (probably FACs) but not downstream of the JA signaling. Besides JA, ET is also one of the main signaling molecules mediating plant defense against herbivores^44^. ET treatment induced extensive transcriptional changes and certain W+OS-elicited *GmCDPK* expression patterns, such as the up-regulation of *GmCDPK23* or the suppression of *GmCDPK*s *5*, *30* and *41* might be dependent on W+OS-elicited ET. Interestingly, MeJA-induced *GmCDPK7* and *GmCDPK30* were both suppressed by ET (Fig. 7C), indicating a potential antagonism of JA and ET in regulating these transcripts. In contrast, the responses of *GmCDPK*s to aphid feeding partly overlapped with SA-induced *GmCDPK* expression. As aphid herbivory elicited high levels of SA (Fig. 6B), we suspect that the SA signaling pathway is involved in aphid-induced transcriptional regulation of *GmCDPK*s.

In addition, CDPKs have been shown to play important roles in plants responses to abiotic stresses, especially ABA and drought^45^,^46^,^47^,^48^. Our analyses revealed that almost 80% of all *GmCDPK*s responded to either ABA, drought treatment, or both, supporting the notion that CDPKs are central regulators of plant drought stress responses. ABA- and drought-induced transcriptional patterns of *GmCDPK* greatly overlap, nevertheless, about half of the drought-induced transcripts were not regulated by ABA, indicating that other regulatory factors are also upstream of the transcriptional regulation of *GmCDPK*s. As expected, drought- and herbivore-induced expression patterns did not show much overlap.

Duplicated genes might undergo different evolutionary processes including nonfunctionalization, neofunctionalization, and subfunctionalization, and these are usually associated with divergence of gene expression profiles of the paralogous genes^49^. Among the 24 paralogous pairs, 3 pairs *GmCDPK10/46*, *GmCDPK8/34* and *GmCDPK23/43* were induced by wounding (Fig. 3A); *GmCDPK23/43* was also induced by W+OS. Three paralogous pairs, *GmCDPK19/47*, *GmCDPK18/44*, and *GmCDPK6/36* were suppressed after W+OS (Fig. 3A); 2 paralogous pairs *GmCDPK5/24* and *GmCDPK6/36* were induced by aphid feeding (Fig. 5); ET increased the expression of the paralogous *GmCDPK9/45* but suppressed *GmCDPK7/35* (Fig. 7C). ABA induced 5 paralogous gene pairs, 3 of which were also induced by drought, and drought stress increased the expression of 10 pairs in total (Fig. 8). In contrast, the expression patterns of two paralogous pairs, *GmCDPK4/26* and *GmCDPK37/41,* differed dramatically. *GmCDPK4* was induced after W+W, W+OS and ABA treatments but the paralog *GmCDPK26* was suppressed following W+OS treatment (Fig. 3A) and induced by drought (Fig. 8). Similarly, W+W and W+OS elicited the expression of *GmCDPK37*, but *GmCDPK41* was suppressed by W+OS (Fig. 3A); *GmCDPK37* tended to be suppressed by SA, while *GmCDPK41* was induced by SA (Fig. 7B). These differences suggest possible neofunctionalization of *GmCDPK*s following gene duplication. Several other duplicated gene pairs (such as *GmCDPK14/21*, *GmCDPK27/48* and *GmCDPK2/30*) demonstrated divergent expression patterns, with one gene responding to the treatment and the other remaining at control levels, indicating that they might have had undergone subfunctionalization. Consistent with the deviating expression patterns of these *GmCDPK* paralogs, in silico promoter analysis indicated that the distributions of *cis*-elements are different between many paralogs’ promoters, probably due to the divergence of the promoters after genome duplication events (Supplementary Dataset 1).

Recent advances have revealed CDPKs as central regulators of Ca^2+^ -mediated immune and stress responses that are crucial for plant survival^50^,^51^. Various CDPKs mediate transient and sustained transcriptional reprogramming and play key roles in the regulation of JA, SA, ET and ABA signaling, facilitating plant responses to wounding, herbivory and drought. Our data shed light on the potential roles of GmCDPKs in regulating soybean responses to biotic and abiotic stresses and provide evidence that many GmCDPKs may have functionally diverged since the two soybean genome duplications. Future genetic and biochemical studies are needed to unravel GmCDPKs’ functions in plant adaptation to environmental factors and the underlying mechanisms.

## Methods

### Identification and structure analyses of CDPKs in soybean and two other legume species

The predicted protein sequences of soybean *Glycine max* were downloaded from Phytozome 9.0 database (http://www.phytozome.net/soybean)^52^, and domains and functional sites in each protein were analyzed with ps_scan.pl^53^. All candidates with protein kinase domains (PS50011) and EF-hand calcium-binding domains (PS50222) were extracted and used to search against the GenBank non-redundant (nr) protein database. The sequences with CDPK-related protein kinases (CRK), calcium/calmodulin-dependent protein kinases (CaMK), or calcium and calcium/calmodulin-dependent protein kinases (CCaMK) as the top hits were filtered out, and the remaining proteins were considered as GmCDPKs. These GmCDPKs were further used as queries to search the draft genome and predicted cDNAs of soybean from Phytozome 9.0 database to obtain potentially overlooked GmCDPKs. The molecular masses were calculated using the Compute pI/Mw tool of ExPaSy (http://web.expasy.org/compute_pi/). The schematic GmCDPK gene structure diagrams were drawn on the Gene Structure Display Server (http://gsds.cbi.pku.edu.cn/)^54^. Myristoylation and palmitoylation sites were predicted using the Plant-Specific Myristoylation Predictor (http://plantsp.genomics.purdue.edu/myrist.html) and the CSS-Palm 3.0 software^55^, respectively. The subcellular localization in chloroplasts and mitochondria was predicted using TargetP (http://www.cbs.dtu.dk/services/TargetP) with the cutoff for specificity set to 90%. Nuclear localization was predicted by NucPred (https://www.sbc.su.se/~maccallr/nucpred). The annotated proteins of two other legume species, *Medicago truncatula* and *Lotus japonicas*, were retrieved from Phytozome 10 database (http://phytozome.jgi.doe.gov) and Kazusa DNA Research Institute (ftp://ftp.kazusa.or.jp/pub/lotus/), respectively. The same procedure was used to identify the CDPKs in these two legumes.

### Phylogenetic analysis of GmCDPK proteins

The putative protein sequences were aligned using the ClustalX2 software^56^ followed by visual inspection. Gaps and ambiguously aligned sites were removed manually. The optimal model with rate heterogeneity was determined by ModelGenerator^57^. Phylogenetic analyses were carried out with a maximum likelihood method using PHYML 3.0. Bootstrap analysis used 100 pseudo-replicates.

### Expression profile based on the estimation of expression levels from RNA-Seq data

The estimated expression levels, RPKM (reads per kilobase per million reads) values, for each *GmCDPK*s from 6 different tissues and developmental stages were obtained from SoyBase (http://soybase.org/soyseq), reanalyzed, log-transformed and visualized using Circos. Samples include young leaves (0.4 Leaflets unfurled), flowers, one cm pod, seeds 14 days after flowering (DAF), roots, nodules, which are described in detail at SoyBase (http://soybase.org/soyseq/tables_lists/index.php).

### Prediction of the GmCDPK promoter cis-elements

The 2 kb regions upstream of the putative translational initiation sites in all the 48 *GmCDPK* genes were retrieved from the soybean genome assembly in the Phytozome 9.0 database (Supplemental Dataset 1) and were analyzed to predict the *cis*-regulatory elements using the PlantCARE^58^ (http://bioinformatics.psb.ugent.be/webtools/plantcare/html/). The motifs putatively involved in transcriptional initiation, transcriptional enhancement, and hormone and/or stress responsiveness were summarized.

### Plant growth, insect rearing, and sample treatments

Seeds of the soybean genotype Huachun No. 6 were grown in the greenhouse at 24 to 28 °C under 16 h of light supplied by sodium lights. All treatments were performed on leaves of the second trifoliate. Soybean aphids, *Aphis glycines*, were from a population maintained on soybean in the greenhouse. For treatments, 10 nymphs and 10 adults were placed at the abaxial site of a second trifoliate leaf and confined in clip cages to avoid moving to other uninduced leaves; empty clip cages were used as control treatment. For the collection of *S. exigua* oral secretions, larvae were reared on soybean until the third to fifth instars. OS were collected on ice using a pipette. For W+W treatments, leaves were wounded with a pattern wheel, and 20 μL of water was rubbed onto each leaf; for W+OS treatments, 20 μL of *S. exigua* OS was rubbed into wounds. MeJA was dissolved in heat-liquefied lanolin at a concentration of 7.5 mg/mL; 20 μL of the resulting lanolin paste was applied to leaves, and pure lanolin was applied as a control. For SA, ET and ABA treatments, 1 mM SA, ACC, or ABA were sprayed on the top and the bottom of each leaf, water was sprayed as control. After specific times, leaves were excised, immediately frozen in liquid nitrogen, and stored at −80 °C until use. For drought treatment, watering was stopped for 5 days and samples were harvested when leaves begun to loose turgor and wilted.

### RNA extraction and q-PCR

Total RNA was extracted from leaves using TRIzol reagent (Invitrogen) following the manufacturer’s instructions. A total of 0.5 μg of total RNA per sample was reverse transcribed using oligo(dT) and Superscript II reverse transcriptase (Invitrogen). q-PCR was performed on a CFX Connect^TM^ real-time system (BIO-RAD) using iTaq^TM^ Universal SYBR Geen Supermix kits (BIO-RAD). For each analysis, a linear standard curve, threshold cycle number versus log (designated transcript level), was constructed using a series dilution of a specific cDNA standard; the levels of the transcript in all unknown samples were determined according to the standard curve. The soybean *actin* gene, which is a housekeeping gene, was used as an internal standard for normalizing cDNA concentration variations. Relative transcript levels of genes were obtained by dividing the extrapolated transcript levels of the target genes by the levels of *actin* from the same sample. Sequences of primers used for q-PCR are listed in Supplementary Table S3.

### Analysis of JA and SA concentrations

One milliliter of ethyl acetate spiked with 200 ng of D_2_-JA and 40 ng of D_4_-SA, the internal standards for JA and SA, respectively, was added to each crushed leaf sample (approximately 150 mg). Samples were then vortexed for 10 min. After centrifugation at 13,000 g for 10 min at 4 °C, the supernatants were transferred to fresh tubes and evaporated to dryness in a vacuum concentrator (Eppendorf). Each residue was resuspended in 0.5 mL of 70% methanol (v/v), vortexed for 10 min, and then centrifuged at 13,000 g for 15 min at 4 °C to remove particles. The supernatants were analyzed on an HPLC-MS/MS (LCMS-8040 system, Shimadzu).

### Statistical analysis

Data were analyzed by unpaired *t*-test using StatView, version 5.0 (SAS Institute).

## Additional Information

**How to cite this article**: Hettenhausen, C. *et al.* Genome-wide identification of calcium-dependent protein kinases in soybean and analyses of their transcriptional responses to insect herbivory and drought stress. *Sci. Rep.* **6**, 18973; doi: 10.1038/srep18973 (2016).

## Supplementary Material

Supplementary Information

Supplementary Dataset
