# Supplementary material for: Genome-wide identification of calcium-dependent protein kinases in soybean and analyses of their transcriptional responses to insect herbivory and drought stress
Source: Sci Rep. 2016 Jan 6;6:18973. doi: 10.1038/srep18973 (PMC4702179; doi:10.1038/srep18973)
Supplement: Supplementary Information [file srep18973-s1.pdf]

Supplementary Figures and Tables

Genome-wide identification of calcium-dependent protein kinases in soybean and analyses of their transcriptional responses to insect herbivory

Christian Hettenhausen, Guiling Sun, Yanbiao He, Huifu Zhuang, Ting Sun, Jinfeng Qi, and Jianqiang Wu

Department of Economic Plants and Biotechnology, Kunming Institute of Botany, Chinese Academy of Sciences, Kunming 650201, China

Yunnan Key Laboratory for Wild Plant Resources, Kunming Institute of Botany, Chinese Academy of Sciences, Kunming 650201, China

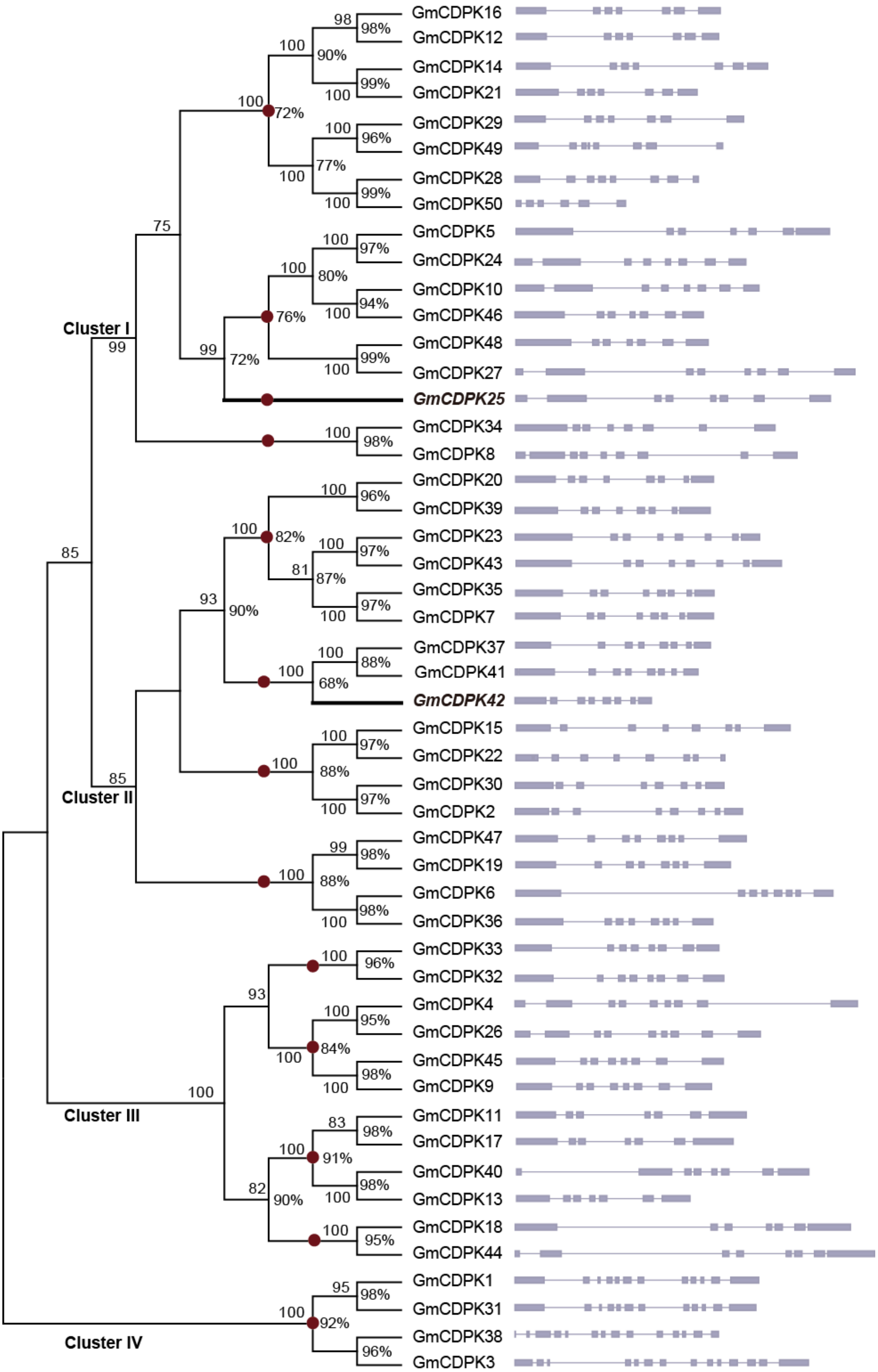
GmCDPK16
GmCDPK12
GmCDPK14
GmCDPK21
GmCDPK29
GmCDPK49
GmCDPK28
GmCDPK50
GmCDPK5
GmCDPK24
GmCDPK10
GmCDPK46
GmCDPK48
GmCDPK27
GmCDPK25
GmCDPK34
GmCDPK8
GmCDPK20
GmCDPK39
GmCDPK23
GmCDPK43
GmCDPK35
GmCDPK7
GmCDPK37
GmCDPK41
GmCDPK42
GmCDPK15
GmCDPK22
GmCDPK30
GmCDPK2
GmCDPK47
GmCDPK19
GmCDPK6
GmCDPK36
GmCDPK33
GmCDPK32
GmCDPK4
GmCDPK26
GmCDPK45
GmCDPK9
GmCDPK11
GmCDPK17
GmCDPK40
GmCDPK13
GmCDPK18
GmCDPK44
GmCDPK1
GmCDPK31
GmCDPK38
GmCDPK3
Cluster I
Cluster II
Cluster III
Cluster IV

Supplementary Figure S1. The phylogeny and gene structures of GmCDPKs.

A maximum likelihood tree of all 50 GmCDPKs was constructed and is shown on the left. The 4 Clusters (I-IV) are indicated at the branches. Numbers above or below branches show bootstrap support values of maximum likelihood analyses. The percentage numbers show the lowest sequence identity values among the homologs. The branches/nodes for GmCDPK25 and GmCDPK42 that potentially lost their paralogous counterparts are marked in bold and italic. Thirteen internal branches are highlighted with brown dots to mark the positions of the ancestral CDPKs that existed before the split of eudicots and monocots. The corresponding gene structure for each *GmCDPK* is shown at the right of their sequence IDs, with boxes and lines indicating exons and introns, respectively. The sizes of exons and introns are proportional to their sequence lengths.

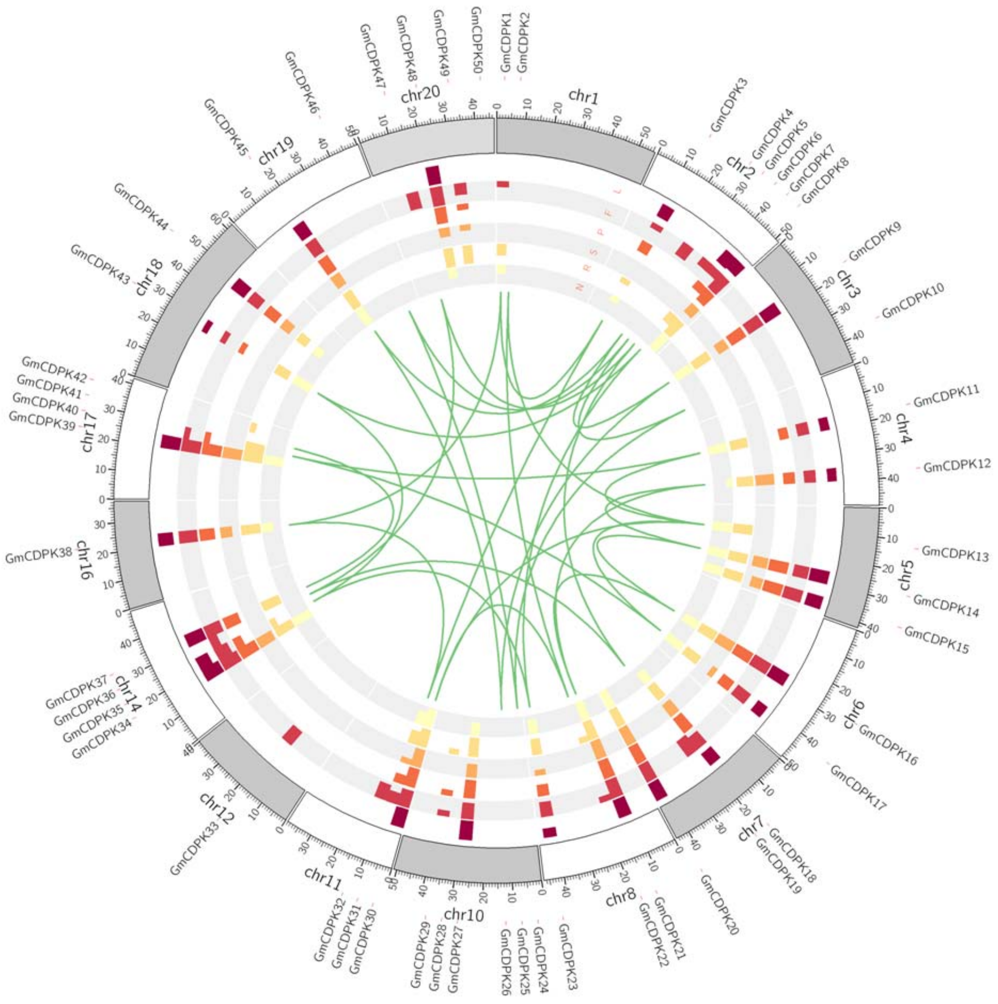

Supplementary Fig. S2. ***GmCDPKs*' Chromosome distribution, development-related expression profile, and paralogous relationships resulted from the early legume genome duplication event.**

The distribution of *GmCDPK* genes on the 17 soybean chromosomes are given in the outer circle, where the numbers represent the chromosome length in Mb. *GmCDPK*s' expression levels in 6 different tissues and developmental stages are displayed as filled blocks in yellow to dark red, with the block height proportional to the expression level ($\log_{10}$ transformed) (L, young leaves (0.4 Leaflets unfurled); F, flowers; P, one cm pods; S, seeds 14 days after

flowering; R, roots; N, nodules). Gene pairs with sequence identities less than 94%, which probably derived from the early genome duplication in legume, are depicted by arcs in the inner circle. The location of GmCDPK genes in the chromosomes is disproportional to the real for the convenience of display.

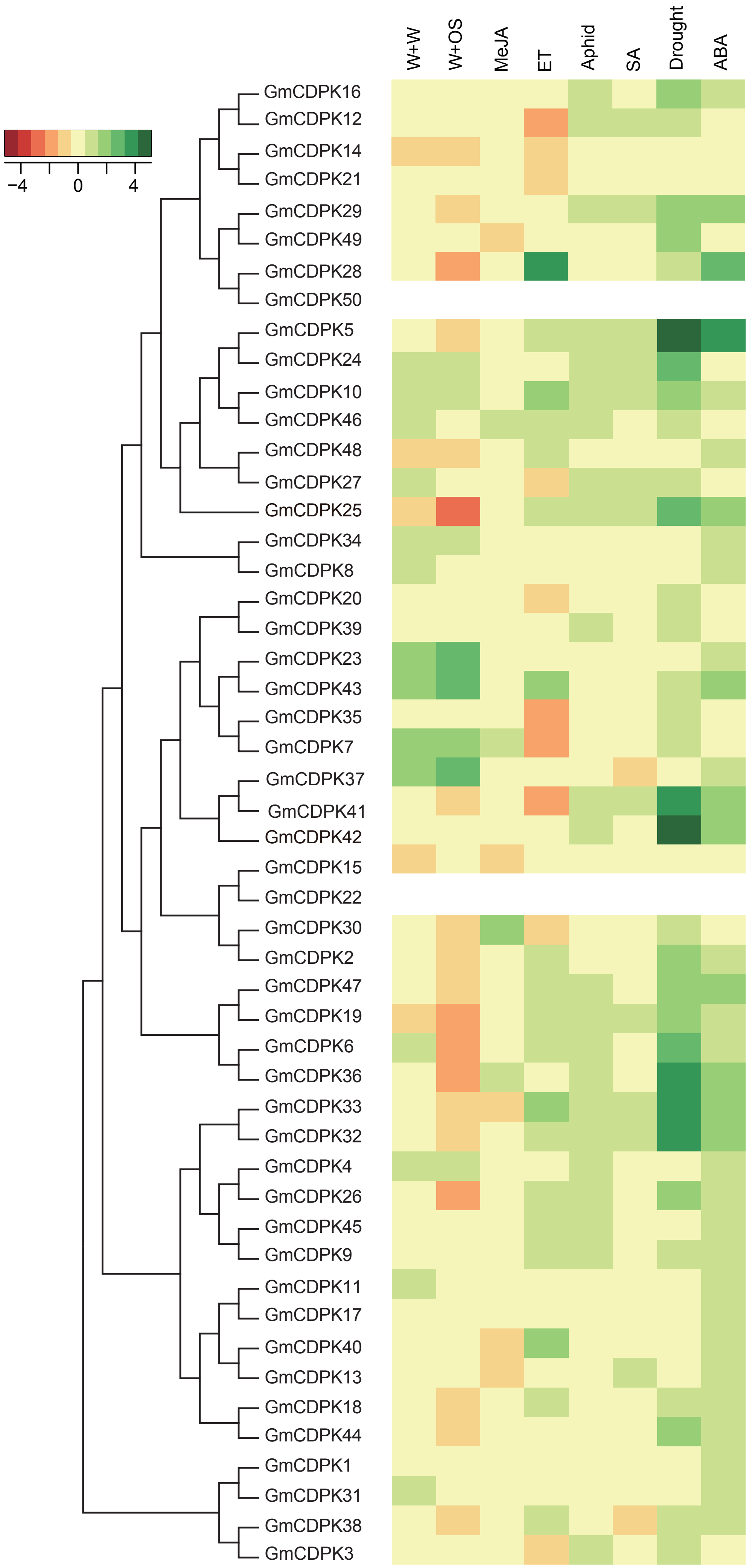

W+W
W+OS
MeJA
ET
Aphid
SA
Drought
ABA
−4
0
4
GmCDPK16
GmCDPK12
GmCDPK14
GmCDPK21
GmCDPK29
GmCDPK49
GmCDPK28
GmCDPK50
GmCDPK5
GmCDPK24
GmCDPK10
GmCDPK46
GmCDPK48
GmCDPK27
GmCDPK25
GmCDPK34
GmCDPK8
GmCDPK20
GmCDPK39
GmCDPK23
GmCDPK43
GmCDPK35
GmCDPK7
GmCDPK37
GmCDPK41
GmCDPK42
GmCDPK15
GmCDPK22
GmCDPK30
GmCDPK2
GmCDPK47
GmCDPK19
GmCDPK6
GmCDPK36
GmCDPK33
GmCDPK32
GmCDPK4
GmCDPK26
GmCDPK45
GmCDPK9
GmCDPK11
GmCDPK17
GmCDPK40
GmCDPK13
GmCDPK18
GmCDPK44
GmCDPK1
GmCDPK31
GmCDPK38
GmCDPK3

Supplementary Fig. S3. *GmCDPK* expression profiles across different treatments.

A maximum likelihood tree of all 50 GmCDPKs was constructed and is displayed at the left. The heat-map on the right shows LOG2-transformed *GmCDPK* expression levels in leaves subjected to 8 different treatments. The truncated and the putatively pseudogenized *GmCDPK22* and *GmCDPK50* were excluded from the analysis.

Supplementary Table S1 Characteristics of the soybean GmCDPKs

Name	Phytozome ID	PACID	Introns	cDNA length	Amino acids	M.W. (kDa)	No. of EF hands	Amino acids at N-terminus	M-Myrist	N-Palmit#	Subcellular localization
GmCDPK1.1	Glyma01g37100.1	26325114	11	2456	562	63.7	4	MGLCFSST	Yes	4,31	Cp
GmCDPK1.2	Glyma01g37100.2	26325115	10	2724	519	58.9	3	MGLCFSST	Yes	4,31	Cp
GmCDPK2	Glyma01g43241.1	26323834	7	1999	542	61.6	4	MRQKPNVR	Yes	No	-
GmCDPK3	Glyma02g05440.1	26289399	13	2522	530	60.1	4	MGACFSAT	Yes	4	Cp
GmCDPK4.1	Glyma02g31490.1	26287928	8	2348	525	60.0	4	MGNCCVLP	Yes	4,5	-
GmCDPK4.2*	Glyma02g31490.2	26287929	8	2223	426	48.5	4	MRHLPKHP	No	No	-
GmCDPK5	Glyma02g34890.1	26288903	7	2448	587	65.6	4	MGNNCVGS	Yes	5	Cp
GmCDPK6	Glyma02g44720.1	26287639	7	2292	527	58.7	4	MGNCCSGG	Yes	4,5	-
GmCDPK7	Glyma02g46070.1	26288212	7	2447	528	59.5	4	MGCCMSKK	Yes	3,4	-
GmCDPK8	Glyma02g48160.1	26288759	8	2507	549	61.5	4	MGNTCRGS	Yes	5	-
GmCDPK9.1	Glyma03g29450.1	26334057	7	2344	534	60.1	4	MGNCCATP	Yes	4,5	-
GmCDPK9.2	Glyma03g29450.2	26334058	6	1977	497	55.7	3	MGNCCATP	Yes	4,5	-
GmCDPK10	Glyma03g36240.2	26335659	6	2429	590	66.5	4	MMGNNCVG	Yes	6	-
GmCDPK11	Glyma04g34440.1	26304777	6	2581	556	62.9	4	MGNCNACA	Yes	4,7	-
GmCDPK12	Glyma04g38150.1	26305524	6	1877	496	55.9	4	MAKPHSGT	No	No	-

GmCDPK13	Glyma05g01470.1	26321134	6	2219	539	61.1	4	MGNCNACV	Yes	4	-
GmCDPK14	Glyma05g33240.1	26322379	6	2061	507	57.1	4	MAAKSSSS	No	50	-
GmCDPK15	Glyma05g37260.1	26320132	7	2194	518	58.3	4	MGNCSSGA	Yes	4	Cp
GmCDPK16	Glyma06g16920.1	26299165	6	1993	497	56.0	4	MSNSTTGT	No	48	-
GmCDPK17	Glyma06g20170.1	26301340	6	2675	551	62.5	4	MGNCNVCA	Yes	4,7	-
GmCDPK18	Glyma07g18310.1	26348115	6	2771	533	59.7	4	MGNCCRSP	Yes	4,5	-
GmCDPK19	Glyma07g36000.1	26349024	7	2339	541	60.3	4	MGNCCSQG	Yes	4,5	-
GmCDPK20	Glyma07g39010.1	26348563	7	2250	529	58.7	4	MGCHGSKE	Yes	3	-
GmCDPK21	Glyma08g00840.1	26309927	6	2223	508	57.2	4	MAAKSSSS	No	51	-
GmCDPK22*	Glyma08g02300.2	26310846	8	1377	458	52.1	4	PPATSSLG	No	No	-
GmCDPK23	Glyma08g42850.1	26309785	7	2582	551	61.8	4	MGCCASKE	Yes	3,4	-
GmCDPK24	Glyma10g10501.1	26346648	7	2677	592	66.2	4	MGNNCVGS	Yes	5	Cp
GmCDPK25	Glyma10g11020.1	26346495	6	2911	595	65.8	4	MGNNCVGP	Yes	5	-
GmCDPK26.1	Glyma10g17561.1	26346683	8	2329	528	60.2	4	MGNCCVVP	Yes	4,5	-
GmCDPK26.2	Glyma10g17561.2	26346684	8	2436	528	60.2	4	MGNCCVVP	Yes	4,5	-
GmCDPK27.1	Glyma10g23620.2	26346632	7	2426	581	65.0	4	MGNTCVGP	Yes	5	-
GmCDPK27.2	Glyma10g23620.3	26346633	6	2669	581	65.0	4	MGNTCVGP	Yes	5	-
GmCDPK28	Glyma10g36090.2	26344103	7	1677	558	63.5	4	MYTSLEIL	No	55	-
GmCDPK29	Glyma10g36100.1	26345626	6	1893	492	55.3	4	MQKHGFAS	No	No	Mt

GmCDPK30	Glyma11g02260.1	26297855	7	2109	505	57.1	4	MGNCNSEP	Yes	4	Cp
GmCDPK31	Glyma11g08180.1	26297646	11	2413	562	63.5	4	MGICFSAT	Yes	4	Cp
GmCDPK32	Glyma11g13740.1	26296164	7	2287	542	61.7	4	MGSCISTQ	Yes	4	Cp
GmCDPK33	Glyma12g05730.1	26294512	7	2282	533	60.6	4	MGSCISTQ	Yes	4	Cp
GmCDPK34.1	Glyma14g00320.1	26285999	7	2622	558	62.7	4	MGNTCRGS	Yes	5	-
GmCDPK34.2	Glyma14g00320.2	26285997	8	2571	558	62.7	4	MGNTCRGS	Yes	5	-
GmCDPK34.3	Glyma14g00320.3	26285998	8	2558	558	62.7	4	MGNTCRGS	Yes	5	-
GmCDPK35.1	Glyma14g02680.1	26286688	7	2344	539	60.7	4	MGCCLSKK	Yes	3,4	-
GmCDPK35.2	Glyma14g02680.2	26286689	4	1697	443	49.5	1	MGCCLSKK	Yes	3,4	-
GmCDPK36	Glyma14g04010.1	26285792	7	2374	529	59.0	4	MGNCCSGG	Yes	4,5	-
GmCDPK37.1	Glyma14g40090.1	26286236	7	1943	526	59.4	4	MGLGMFKA	No	11,12	-
GmCDPK37.2	Glyma14g40090.2	26286235	7	1998	537	61.0	4	MGHCFSKP	Yes	4	Nuc
GmCDPK38	Glyma16g23870.3	26352812	13	1665	554	62.8	4	MSREGYHP	Yes	30	Cp
GmCDPK39	Glyma17g01730.1	26313594	7	2506	538	59.8	4	MGCQGSKE	Yes	3	-
GmCDPK40	Glyma17g10410.1	26314025	7	2386	541	61.4	4	MGNCNACV	Yes	4,7	-
GmCDPK41	Glyma17g38040.1	26311092	8	2017	583	66.9	4	MILFFVSE	Yes	67	Nuc
GmCDPK42	Glyma17g38050.2	26312864	7	1825	513	58.3	4	MKMGVGFS	No	56	-
GmCDPK43.1	Glyma18g11030.1	26355363	7	2789	551	61.9	4	MGCCVSKD	Yes	3,4	-
GmCDPK43.2	Glyma18g11030.2	26355362	8	2729	551	61.9	4	MGCCVSKD	Yes	3,4	-

GmCDPK43.3	Glyma18g11030.3	26355364	8	2713	551	61.9	4	MGCCVSKD	Yes	3,4	-
GmCDPK44	Glyma18g43160.1	26356996	7	2493	512	57.9	4	MGNCCRSP	Yes	4,5	-
GmCDPK45	Glyma19g32260.1	26327943	7	2310	535	60.3	4	MGNCCATP	Yes	4,5	-
GmCDPK46.1	Glyma19g38890.1	26327776	5	2280	560	63.3	3	MMGNNCLG	No	6	-
GmCDPK46.2	Glyma19g38890.2	26327775	6	2600	593	67.0	4	MMGNNCLG	No	6	-
GmCDPK47	Glyma20g08140.1	26337815	7	2403	546	60.9	4	MGNCCSQG	Yes	4,5	-
GmCDPK48.1	Glyma20g17020.1	26337739	7	2322	579	65.0	4	MGNTCVGP	Yes	5	-
GmCDPK48.2	Glyma20g17020.2	26337738	7	2400	579	65.0	4	MGNTCVGP	Yes	5	-
GmCDPK49	Glyma20g31510.1	26338820	7	1443	480	54.0	4	MQKHGFAS	No	No	Mt
GmCDPK50*	Glyma20g31520.2	26337349	5	966	294	33.1	4	MAPEVLRK	No	No	-

"*" indicates that the 5' terminus of the sequence is incomplete. "#" indicates the predicted amino acid sites of palmitoylation in the N-terminal region. Subcellular localization was predicted for the localization in chloroplasts (Cp), mitochondria (Mt), and the nucleus (Nuc).

Supplementary Table S2 Gene IDs for CDPK genes in *Medicago truncatula* and *Lotus japonicus*.

The annotated proteins of two other legume species, *Medicago truncatula* and *Lotus japonicas*, were retrieved from Phytozome 10 database (http://phytozome.jgi.doe.gov) and Kazusa DNA Research Institute (ftp://ftp.kazusa.or.jp/pub/lotus/), respectively.

Medicago truncatula		*Lotus japonicus*	
Gene ID	CDPK Names	Gene ID	CDPK Names
Medtr3g098070.1	MtCDPK1	chr4.CM0004.1050.r2.a	LjCDPK1
Medtr3g051770.1	MtCDPK2	chr4.CM0026.550.r2.m	LjCDPK2
Medtr3g098090.1	MtCDPK3	chr1.CM0029.690.r2.d	LjCDPK3
Medtr8g099095.1	MtCDPK4	chr1.CM0094.80.r2.m	LjCDPK4
Medtr8g095440.1	MtCDPK5	chr2.CM0102.520.r2.d	LjCDPK5
Medtr7g068710.1	MtCDPK6	chr4.CM0119.200.r2.m	LjCDPK6
Medtr7g091890.1	MtCDPK7	chr2.CM0308.470.r2.m	LjCDPK7
Medtr7g106710.1	MtCDPK8	chr6.CM0489.230.r2.d	LjCDPK8
Medtr5g009830.1	MtCDPK9	chr6.CM0553.410.r2.d	LjCDPK9
Medtr5g092810.1	MtCDPK10	chr1.LjT08C17.20.r2.m	LjCDPK10
Medtr5g089320.1	MtCDPK11	chr5.LjT12G17.60.r2.m	LjCDPK11
Medtr5g089440.1	MtCDPK12	chr4.LjT12N18.80.r2.d	LjCDPK12
Medtr5g099240.1	MtCDPK13	chr5.LjT34O04.70.r2.d	LjCDPK13
Medtr5g022030.1	MtCDPK14	chr2.LjT42A12.60.r2.m	LjCDPK14
Medtr0028s0170.1	MtCDPK15	LjSGA_022365.1	LjCDPK15
Medtr1g052530.1	MtCDPK16	LjSGA_045515.1	LjCDPK16
Medtr1g096490.1	MtCDPK17	LjSGA_026936.1	LjCDPK17
Medtr1g041150.1	MtCDPK18	LjSGA_029957.1	LjCDPK18
Medtr1g055255.1	MtCDPK19	LjSGA_014141.2	LjCDPK19
Medtr1g054865.1	MtCDPK20		
Medtr1g026190.1	MtCDPK21		
Medtr4g107490.1	MtCDPK22		
Medtr4g132070.1	MtCDPK23		
Medtr4g066660.1	MtCDPK24		
Medtr4g132040.1	MtCDPK25		

Supplementary Table S3 List of primers used for q-PCR analysis

GmCDPK	Forward primer (5'-3')	Reverse primer (5'-3')
1	CATTATTCCTTTGATCTCTCTCCCA	TTGCGGTTACGGTTGGATGA
2	AATGCGTCAAAAACCGAACG	GAAACGGTGTCGTTACTGCG
3	TGCATCTTTGAGGCGTTTTCG	TGCGCTTTTTGTTGACGTTGA
4	AGAGCCTTGAGGGTGATTGC	AACCCTACACGCAGCTCATC
5	AGCTGGTACCACAAAGGGTT	GTGCTGAGACAAGGGCATCA
6	ACCCTTCCAATGCGACTCTT	TGTTTGACGTTGGGAGACGA
7	AGTTGACTCGGTTCCACCTT	GCCAAAGTTTGATGCAGAATGG
8	GGCAGGAGAGGAGCCAATAC	TCTCCGACAGATCTCCACAA
9	TGCGAAACAAGTTTGACCCTC	AGGGTTTTTGGGGATGGATGT
10	TCTTCGTCCATTTTCGTTTCCA	ATTTCTGCACCAACCCCACT
11	GCCGGAGAAATCAGAACCCA	AGGCGTTGCAGTTTCCCATA
12	ACCGAGTCTTCCTCTGTTTCT	TGTGAGGCTTCGCCATTGAT
13	GCTGCAGGCCTCAAAATGTC	TCACACCGCCAATGAGACAA
14	ATAGCACGCCGAAAAACACG	GATTTCGCCGCCATTGTTGT
15	TTGGCTGCAGAGGTACAGAG	CAACCGCACTTGGGAGACTA
16	GCAGGTCTGGGTCTTCTTTT	TTCTCGGTTCGGTATGGGAG
17	CCGGCGTCACCATACGATAA	TCTACCACGTCAGCTTTCGC
18	ATGCAACACTTCGTTGCGTG	TGGGTCAAATACCTGAGTCAAATCT
19	ACTAATTAATCACCACCCTTCCCA	AGGGAGGGAGGAAAACCACA
20	TGCAAGTGTCTTATCGAAGCCT	GCTTGCACTTCCATATACGGGT
21	AGCTCCACGAAGATACCTCCT	GGGCTGGTCACGTTAGACTT
23	TAAAGTATGTGAATGAACTCCACCT	AAGTGGAGCCTTGAAAGAGGG
24	ACAACTGTGTTGGATCGAGAACT	TCCGCAGCTTTGGTTCTCTT
25	AGCTTTGGTTTAATGATGTTTCACT	GCGGCATTGCATCTCATGATT
26	AGCTCATCGTGCCTTCCTTTT	GGTCCGGCTTCTCCTTCTT
27	TCTGATTTGTACTCTTTTGATGCCA	GGTGCTAAGTTAGTGAAAAGAACA
28	CACGAGACTCCGAACGTGAA	GCTTCAACAGCTTCGCCTTT

29	AGGAAGGCAATTCAATTCGGA	GATACGGCAACACGTTACGC
30	TGCACGACACCTTATCCATCAA	CTGCAACTGCTGCTTCTTGC
31	TCCTTTGATCTCTCTGCCATTC	AGACCTTTGCGGTTACGGTT
32	GGCCTCTCGCCAGTATTCAA	AACTGAGAACTTTGGCCCCT
33	AGAAAATGGCGGGACCAACT	AATTGCGAAGAGGGTGATCG
34	ACGCCAAGAGAGAAGCCATC	GGCCCTGTCCCAATTTACGA
35	CCCACAGTTGACTCGGTTCT	AGGAGAAGCCAAAGTTTGATGC
36	ACCTTTGAACTGAGTTGTTCCA	TTCGGCGAGTCTCTTGCTTT
37	AGCTCCGGAGGTGTTAAAGC	TTCTCTCGTTTTCGCCCCAA
38	GTATCATCCGAACCCCGTCC	CCTTGCGGTTCTTGTTGACG
39	TGCACTACCCTTTCCTTTCCT	AGCAGCAGCTACTGTCCAAG
40	ATGCTGCAGGCCTCAAAATG	AAGTTCGATCACACCGCCAA
41	TGAGGACCATCCCATCCCAT	AGCTTCAGAGTTTGAGAGGTG
42	CTGGCATGAAAATGGGAGTGG	AGTGGAAGTGGAGGTGCAAT
43	ATCTTCCACAATCATCCTTGCAT	GTGGAGCCTTGAAAGAGGGT
44	TTCTGGCAGTGCATGAGGTT	TGCAGTTGCACTCACATTGC
45	GGAAGACGAAGTCTGAGCGA	TGAGAAGGGTTTTGGGGATGG
46	CAACGCGAACAATTCCCACT	ATGGACGAGGAAAAGAGGTGT
47	GCCATTGCCATAATGGAGGG	GTATCCCCTCGCAACCAACA
48	TTGAAAAGAGATATCTGGTGGAGT	AACCATCATTTCAGGTGTTTGC
49	TACTAAGTGTAACCCTTGGGTTGTT	TGAAAGCCTCTCTGCTATGACC
